# Trusted traffickers and prosocial humanities: exploring Ukrainian student vulnerabilities to wartime cross-border risks

**DOI:** 10.3389/fpubh.2026.1818656

**Published:** 2026-10-30

**Authors:** Elina Paliichuk

**Affiliations:** 1 Faculty of Romance and Germanic Philology, Department of Linguistics and Translation, Borys Grinchenko Kyiv Metropolitan University, Kyiv, Ukraine; 2 CEFRES (French Research Center in Humanities and Social Sciences), Prague, Czechia; 3 GAHTS (The Global Association of Human Trafficking Scholars), Toledo, OH, United States

**Keywords:** cognitive linguistics, corpus analysis, education, empirical studies, human trafficking, humanities, Ukraine, war and migration

## Abstract

This paper focuses on students’ potential vulnerability to trust in individuals who might act as facilitators of human trafficking or trafficking-adjacent fraud. The methods used are corpus analysis, conceptual (frame) analysis, and a reading intervention. The media corpus of 115,973 words was used for frame analysis to reveal traffickers’ static and dynamic characteristics. A single-group two-stage pre−/post-reading study involving 69 B.A. students from Borys Grinchenko Kyiv Metropolitan University was conducted using eight media fragments. SPSS Software for Windows 26 was used to analyze the empirical data. Paired-samples analysis showed significantly lower post-reading trust in volunteers, agency representatives, prospective employers, and relatives or friends (V2–V5, V8), whereas trust in loving partners and several categories of unfamiliar people offering help (V6–V7, V9-V10) did not change significantly. These findings are interpreted as within-participant changes in self-reported trust. The results identify trust as a marker of one potential component of trafficking-related vulnerability and support further controlled research into linguistic and educational strategies for primary prevention to address stable patterns of misplaced trust associated with trafficking recruitment tactics. The study contributes to primary prevention through prosocial humanities for protective awareness.

## 1 Introduction

Human trafficking is a global scourge with a complicated criminological structure aggravated by social upheavals and regional economic, political, and cultural factors. In Eastern Europe, the most conspicuous situation in this regard can currently be attributed to Ukraine, whose population is heavily exposed to the grim realities of the aggressive war of the Russian Federation. The massive flows of refugees in the very early days of the war provoked increased risks of trafficking in human beings amid the preexisting socioeconomic trends and vulnerabilities within the country. According to the research by the International Organization for Migration (1), “trafficking in persons is indirectly related to armed conflict when traffickers are targeting victims who are in vulnerable situations due to the conflict”. For instance, displaced persons can be intercepted while fleeing the crisis or trafficked at their destination for domestic servitude, forced labour, or sexual exploitation (1). Traffickers were observed to be “trolling border areas trying to lure refugees with promises of accommodation, onward transportation, or employment” (2).

It is reported that “of those who have fled the country, 90 percent are estimated to be women and children. At the same time, men aged 18-60 are forbidden to leave the country for their duty to serve in the army” (1). Female students and mothers with children may become targets for traffickers thriving on the human tragedy, which preconditions a more resonant gender perspective in the studies on prevention and combating human trafficking in the background of the preexisting vulnerabilities. This gender perspective is interrelated with other vulnerabilities. Trafficking vulnerability is shaped by the interaction of gender with age, disability status, social class, and migration or protection status (3, 4). Age intersects with gender in that younger women and unaccompanied minors face qualitatively different recruitment tactics than older women travelling with dependents (5). Social class and pre-war socioeconomic precarity shape which forms of assistance, e.g., transport, housing, employment, a displaced person is compelled to accept, and from whom. Migration and protection status is itself an aggravating factor: the International Organization for Migration’s Determinants of Migrant Vulnerability model identifies legal status, and specifically the temporariness or precarity of that status, as a factor of vulnerability independent of individual characteristics (6, 7). For the population addressed in this study, this means that a Ukrainian woman student displaced under temporary protection status, potentially travelling with a disability or with dependent family members, and having limited household resources, occupies a materially different risk position than the aggregate “90% women and children” statistic conveys. Therefore, pedagogical and policy implications of this study should take this differentiation into account, as gender is not a uniform category of risk. Such socioeconomic factors as willingness to get a higher income, belief in the idea of a better life abroad, hope of marrying a foreign fiancé, education prospects, and career opportunities stem from embedded patterns of Soviet-like thinking regarding job opportunities and overall life satisfaction. On the other hand, the younger generation^1^ aspires to the free movement of persons, education, and travelling opportunities. The need for security and safety, combined with socioeconomic factors, has predetermined more intense flows of young people abroad.

Human trafficking in Ukraine predates the full-scale Russian invasion and should therefore be understood against a longer history of labour migration, socioeconomic vulnerability and cross-border exploitation. Earlier research described Ukraine as a country of origin, transit and destination for trafficking and documented changes in the profiles and forms of exploitation affecting Ukrainian victims. These earlier patterns are important because the risks generated by the current war interact with pre-existing migration and exploitation structures rather than creating trafficking as an entirely new phenomenon. (8)

Russia’s full-scale invasion in February 2022 nevertheless transformed the scale and conditions of population displacement. Early research consequently warned that forced displacement, family separation, socioeconomic stress, disrupted services and reduced access to reliable information could create or intensify opportunities for trafficking and exploitation. Cockbain and Sidebottom (9), drawing on an international evidence-gathering roundtable involving 103 participants from 65 organisations, identified conflict-related displacement, information gaps, insecurity and constrained access to support as mechanisms capable of increasing trafficking vulnerability. Importantly, however, they also cautioned against overstating the scale of trafficking in the absence of reliable prevalence data, arguing for a distinction between evidence of heightened risk and evidence of trafficking itself.

Subsequent research has reinforced this distinction. Mendel and Sharapov (10), using practitioner survey data, document and media analysis, published research and Eurostat data, found that the large-scale increase in trafficking initially feared after February 2022 had not materialised in the available identification statistics. They attribute this outcome partly to the comparatively extensive rights granted to Ukrainians, particularly legal mobility, access to employment, welfare and public services under temporary protection arrangements. Their findings suggest that reducing dependence on irregular migration and precarious intermediaries can function as a structural form of trafficking prevention. At the same time, they emphasise that the absence of a large increase in formally identified trafficking should not be confused with the absence of exploitation, particularly in labour markets.

Recent quantitative research provides further evidence for this more differentiated picture. The Mixed Migration Centre (11) study of people who fled Ukraine to Berlin, Bern and Warsaw combined 1,602 surveys with 57 key-informant interviews. Six per cent of respondents (104/1,602) reported some form of workplace abuse, while 2% (35/1,602) reported either personally experiencing or observing working conditions that could indicate potential forced-labour trafficking. A smaller proportion reported possible indicators of forced sexual exploitation. At the same time, substantial structural vulnerabilities remained: financial pressure, limited access to decent employment, language barriers, insecure housing and difficulties obtaining reliable information about labour rights were among the factors identified as potentially increasing susceptibility to exploitation.

Sharapov’s (12) study of Ukrainian refugees in Poland and Romania similarly cautions against treating vulnerability and victimisation as interchangeable categories. It conceptualises trafficking vulnerability as the interaction of personal, situational and contextual factors and emphasises that these factors change over time. The initial protection response, including legal mobility pathways, temporary protection and continued functioning of Ukrainian state institutions, appears to have reduced some immediate risks. However, prolonged displacement can generate what the study terms “delayed vulnerability,” as savings are depleted and difficulties in securing employment, housing and services accumulate. Within this study, “trust” is seen as a marker of vulnerability.

Taken together, existing research therefore presents a more complex picture than a simple wartime increase in trafficking numbers. Russia’s full-scale invasion has dramatically expanded and transformed the conditions under which trafficking vulnerability can develop, while available evidence does not demonstrate a proportionate surge in formally identified cases. Moreover, identified-victim statistics cannot be treated as direct measures of prevalence because trafficking is a hidden and underreported phenomenon and because recognition and identification themselves present methodological difficulties. Recent research consequently increasingly examines vulnerability, exploitation and protective factors alongside trafficking statistics.

This distinction is central to the present study. The study investigates one narrower cognitive and behavioural component of vulnerability: “self-reported trust in individuals occupying roles that may be used in trafficking recruitment or trafficking-adjacent deception”. The research therefore asks whether exposure to trafficking-related media narratives is associated with measurable changes in protective caution toward such actors.

Before turning to the conceptual and pedagogical dimensions of this study, it is necessary to define the notion of “human trafficking” from a regulatory perspective, since the term is frequently used loosely in media and public discourse, including, at times, as a near-synonym for fraud or deception. In international law, human trafficking is defined by the Protocol to Prevent, Suppress and Punish Trafficking in Persons, Especially Women and Children, supplementing the United Nations Convention against Transnational Organized Crime [the Palermo Protocol, (13)], which structures the offence around three cumulative elements: an *act* (recruitment, transportation, transfer, harbouring, or receipt of persons), a *means* (threat or use of force, coercion, abduction, fraud, deception, abuse of power or of a position of vulnerability, or the giving or receiving of payments to achieve control over a person), and a *purpose* (exploitation, including at minimum the exploitation of the prostitution of others, other forms of sexual exploitation, forced labour or services, slavery or practices similar to slavery, servitude, forced criminality, or the removal of organs) (14, 15). Where the victim is a child, the means element is not required for the act to constitute trafficking [(13), Art. 3(c)]. Therefore, the said act-means-purpose structure serves as the distinctive feature of trafficking, as any single deceptive exchange does not constitute trafficking yet belongs to other border-crossing offences.

This distinction matters because trafficking is routinely conflated with migrant smuggling in both media and lay perception. Under the Protocol against the Smuggling of Migrants by Land, Sea and Air (14), smuggling is defined as the procurement of illegal entry into a state for financial or material benefit and characterised by the migrant’s consent and by a transactional relationship that typically ends on arrival (16). Trafficking, by contrast, does not require an international border crossing, is defined by exploitation rather than by the illegality of entry, and renders the victim’s apparent consent legally irrelevant once coercion, deception, or abuse of vulnerability has been used to obtain it [(14), Art. 3(b); (17)]. In practice, the two phenomena often overlap and evolve into one another; for example, a person who initially consents to being smuggled may subsequently be trafficked.

The Recommended Principles and Guidelines on Human Rights and Human Trafficking (18) further define trafficking as, at once, a criminal offence and a human rights violation, requiring that “the human rights of trafficked persons shall be at the centre of all efforts to prevent and combat trafficking” ((18), Principle 1). This human rights-based framing is supported in Directive 2011/36/EU of the European Parliament and of the Council on preventing and combating trafficking in human beings, which extends the exploitation spectrum to include forced criminality, forced begging, and forced marriage, and which frames trafficking, in its first recital, as a violation of human rights and of human dignity (19). Framing trafficking as a human rights violation carries direct implications for state responsibility. The European Court of Human Rights held that trafficking falls within the scope of Article 4 of the European Convention on Human Rights (prohibition of slavery, servitude, and forced labour), and that this provision imposes positive obligations on states: to adopt an adequate legislative and administrative framework, to take protective operational measures once authorities knew or ought to have known of a real risk to a specific individual, and to investigate credible suspicions of trafficking, including across borders (20). This due diligence standard is supported in the OHCHR Recommended Principles’ requirement that states act with due diligence to prevent trafficking, protect victims, and prosecute offenders [(18), Principle 2] means that awareness-raising of the kind piloted in this study is, at most, one contribution to a much larger structure of state responsibility. And the present paper’s findings are discussed accordingly in the sections that follow.

According to the Law of Ukraine “On Counteracting Human Trafficking” (21), local self-government bodies, enterprises, institutions, organisations, and individual citizens shall implement measures to prevent and combat human trafficking. With the outbreak of the full-scale Russia-Ukraine war, the safety of Ukrainian citizens has changed dramatically due to human trafficking risks soaring because of forced migration. For the Ukrainian wartime context specifically, the exploitation spectrum relevant to displaced populations spans sexual exploitation, forced labour, domestic servitude, forced criminality, and, for men of conscription age who remain in-country, coerced participation in armed war-adjacent activities in the occupied territories, each of which is a distinct, legally cognisable form of exploitation.

This legal act-means-purpose grounding is represented in the frame model developed in this paper (see Conceptual portrait of trafficker below). The types of individuals who might serve as facilitators of traffickers identified through corpus analysis (the volunteer, the agency representative, the loving partner, the employer) are not equally positioned with respect to criminal liability. Whether a given individual represents a *trafficking risk* (i.e., a facilitator whose conduct meets the act-means-purpose threshold of Article 3) or a *fraud risk* (an individual engaged in deception without a subsequent nexus to exploitation) is not always resolvable from short media excerpts, and the present study does not claim to adjudicate this for each case. Rather, the frame model is used descriptively to capture how these individuals are *linguistically constructed* in media discourse, predetermining social beliefs, stereotypes, and behaviours. This introductory section seeks to establish the legal threshold against which such linguo-conceptual constructions should ultimately be read.

The distinctive features of traffickers should be given fair attention to facilitate the recognition of danger and stimulate critical thinking among potential victims. Media red-flagged cases^2^ of sexual exploitation and bonded servitude committed by individuals who deceptively presented themselves as hosts, volunteers, employers, or social workers, performing the role of a “good Samaritan” as a recruitment tactic (67).^3^ Under extreme conditions, such as fleeing war, vulnerability may include faster acceptance of help or the destruction of protective cognitive borders, resulting in one’s trust and belief in sincere help, love, friendship, etc. Therefore, awareness activities should include the exposure of vulnerable social groups to a comprehensive portrait of the trafficker.

It should be noted that over the last decades, the studies on human trafficking have shifted the focus from the statistical description to more victim-oriented approaches through survival narratives and analysis of the vulnerabilities (22–25). Such studies deal mostly with the issues of early detection and rehabilitation from the point of view of social health, the judicial realm, criminology, economic, political, or cultural prerequisites, psychology of vulnerability, etc. The role of education in combating human trafficking is mainly allotted to non-governmental organisations through the informative activities held in educational establishments by disseminating booklets, delivering special training, discussing movies or stories, and other forms of interaction with young people. However, there is a need for a systematic highlighting of the risks of human trafficking, which can be done in the form of regular activities included in humanities.

This study uses the term *prosocial humanities*, where “prosocial” is used in its established sense from social psychology: voluntary behaviour intended to benefit another person or group (26). Applied to humanities pedagogy, prosocial humanities refer to the deliberate orientation of literary, linguistic, and stylistic instruction toward a social-protective outcome, which is trafficking-risk awareness in this case. It is not directed toward literary appreciation, linguistic competence, or textual analysis skills as a final competence. Such an approach differs from standard humanities or stylistics education, in which close reading and stylistic analysis are typically taught as disciplinary competencies. In the prosocial humanities framing adopted here, the analytical skills developed through close reading [following (27, 28)] are seen as part of a specific protective outcome, and the curriculum is designed and evaluated accordingly.

The development of socially oriented humanities courses^4^ should be based on sound research on students’ vulnerabilities and centered on methods of acquiring the skills to identify the signs of traffickers. This paper focuses on exploring student presuppositions to trust individuals at the border crossing, some of whom might be involved in human trafficking schemes, in the context of the Russia-Ukraine war, based on the reading interventions conducted at a Ukrainian university. It suggests exploring students’ vulnerabilities through pre- and post-reading surveys. This approach presupposes analytical reading activities in Stylistics and meets the needs of revealing the types of individuals whom students are more inclined to trust at the pre-reading stage, measuring reactions to the texts profiling specific types of traffickers at the post-reading stage, and fostering metalinguistic reflection and discussion at the post-survey stage, which may contribute to deeper processing, understanding the risks, and memorability of the cases described in media texts.

The research is carried out in the framework of the optional component of the course on the Stylistics of the English language as a foreign language through analytical reading of media texts. The reading interventions were conducted within such thematic modules as media style and narrative stylistics, suggested in the curriculum as classes on critical thinking skills and analysis of the media texts. The prosocial perspective was underpinned by cognitive linguistics: frame modelling was done with a focus on the conceptual portrait of the trafficker as a main actor in the human trafficking situation represented in the media. The research aim was to establish student vulnerabilities to trusting individuals potentially involved in human trafficking or in fraudulent schemes that expose them to trafficking risk. The main research question was what types of such individuals were at the pre-reading stage and whether there were significant differences in student perceptions of potential traffickers after they read selected media texts. The null hypothesis presupposed the absence of changes in student perceptions after reading texts about human trafficking risks at the border crossing and, consequently, stable pre-existing vulnerabilities, invoking further studies of each vulnerability. The alternative hypothesis presupposed significant changes in the perceptions of the individuals who may potentially be involved in human trafficking after reading texts about human trafficking risks. It suggested that targeted reading of selected texts could be used for warning interventions in prosocial teaching of humanities at a university.

To find out the dependencies on the reading media texts and changing attitudes to the risks of human trafficking, a set of research tasks have been completed: to explore the corpus of media texts with a focus on the image of the trafficker, to conduct a conceptual analysis and reveal basic types and characteristics of the traffickers, to reveal the preliminary vulnerabilities, to select target texts as materials for the survey, to elaborate questionnaires, to trace post-reading differences in student perceptions of the individuals who may be involved in human trafficking schemes, to interpret the results concerning the hypotheses stated above, and to plan further steps of the implementation of the research in education processes. The paper further provides an observation of the content on human trafficking in the corpus of texts from the media, an overview of the static and dynamic characteristics of traffickers established in the corpus, a description of the reading intervention design and procedures applied to meet the research objective and results of the pre-and-post reading surveys, a discussion of the work within the multidisciplinary paradigm, and the summary of the major findings of this study.

## 2 Methods

The research objective and tasks are achieved through several methodological approaches, including corpus analysis, frame modelling as part of conceptual analysis, and an empirical study in the form of a reading intervention. The study is done in three stages:

- corpus analysis
- conceptual analysis
- the reading intervention is based on the following procedures: pre-reading survey, the selection of texts for targeted reading, post-reading survey, and interpretation of the results.

The combination of these methods proved appropriate for meeting the objectives at each stage subordinate to the main aim of revealing the extent of student vulnerability to trusting in individuals who can be involved in human trafficking while helping Ukrainian refugees at the border crossing.

### 2.1 Corpus analysis

In the stage of the corpus analysis, preparatory work was done to make observations of how human trafficking was reported in the media. To this end, a corpus of 115,973 words (4,917 sentences) from the media 2015–2024 was compiled^5^ in the Sketch Engine text analysis software. The language dataset was (64) annotated under the keywords associated with the human trafficking topic. Attention was given to the verbal representations of TRAFFICKER and other actors who may be involved in the trafficking activities^6^.

### 2.2 Conceptual analysis

The observations of the corpus were followed up with the conceptual analysis based on the theory of five frames by Pr. S. Zhabotynska’s methodology (29). It assumes that each conceptual structure is predetermined by a limited set of propositions^7^ where basic categories of thought are arranged by the way we perceive things in the experiential world [(29): 80]. Those are the Thing Frame, Action Frame, Possession Frame, Identification Frame, and Comparison Frame, each having iterative propositional schemas. To reveal the static characteristics of traffickers, the following frame models were used in the study:

- the Thing Frame for modelling traffickers’ *qualitative, quantitative, locative, temporative*, and *mode of existence* propositions,
- the Possession Frame, for tracing the *part-whole, inclusion,* and *ownership* propositions,
- The Identification Frame for revealing *personification, classification*, and *characterisation* propositions,
- the Comparison Frame for analysing the relations of *identity, similarity*, and *likeness*^8^.

The Action Frame was modelled to reveal the dynamic characteristics of *traffickers* as *actors* in human trafficking situations through the *state, process, contact, causation, agent-affected, result*, and *consequence* propositions.

The technique of frame modelling used in this study established the core semantic roles assigned to *traffickers* in media representation, including their types and static and dynamic characteristics. The static characteristics are the typical descriptive features of traffickers, while the dynamic ones actuate the most frequent actions against the victims or potentially vulnerable groups. Taken together, the five frames show a comprehensive conceptual portrait of traffickers which can serve as a springboard for developing preventive educational strategies.

Throughout this paper, the actors modelled through frame analysis (volunteer, agency representative, employer, relative/friend, loving partner, stranger) are discussed in terms of the *risk* they represent to a person moving abroad. Since “risk” is used loosely in media discourse, three categories are operationalised here, following the elements of the Palermo Protocol (14) and the ILO’s operational indicators of trafficking (30):

Trafficking risk is present when a trafficker’s conduct satisfies the act–means–purpose structure of Article 3 as an act of recruitment, transport, transfer, harbouring, or receipt; achieved through a means involving force, coercion, abduction, fraud, deception, or abuse of a position of vulnerability; for exploitation (sexual exploitation, forced labour, servitude, forced criminality, or organ removal). Consent obtained through any of the listed means is legally irrelevant. A cross-border element is not required.

Smuggling risk is present when a trafficker facilitates illegal border crossing for financial or material benefit, where the person’s consent is freely given, and the relationship is transactional and typically concludes on arrival, with no subsequent exploitation [UN, 2000, Smuggling Protocol; (16)]. A cross-border element is definitionally required.

General fraud risk is present when a trafficker deceives a person for material gain (e.g., false promises of employment, fees for non-existent services, non-delivery of agreed terms) without evidence of a subsequent exploitative purpose. Consent is vitiated with respect to the specific deception, but there is no coercive control over the person’s subsequent labour, body, or movement. No cross-border element is required.

These three categories are not mutually exclusive over time: a case may begin as apparent fraud or smuggling and evolve into trafficking once exploitation begins. The differentiating parameters used to classify corpus and survey material in this study are summarised in Table 1. Differentiating parameters across risk categories (see Supplementary Material).

**Table 1:** Paired samples: pre- and post-reading responses (*N* = 69).

Variable	Item	Pre-reading mean	Post-reading mean	Mean difference	*p* (one-tailed)	Interpretation
V2	Volunteer at the border crossing	2.54	1.99	0.55	<0.001	Significant decrease
V3	Agency representative	2.77	2.41	0.36	0.007	Significant decrease
V4	Prospective employer	2.57	2.00	0.57	<0.001	Significant decrease
V5	Relative or friend	3.54	2.84	0.70	<0.001	Significant decrease
V6	Loving partner or fiancé(e)	3.38	3.20	0.17	0.070	No significant change
V7	Random person who seems kind and willing to help	1.65	1.51	0.14	0.121	No significant change
V8	Young man or woman willing to help	1.64	1.46	0.17	0.079	No significant change
V9	Middle-aged woman offering help	1.78	1.55	0.23	0.039	Significant decrease
V10	Middle-aged man offering help	1.49	1.41	0.09	0.225	No significant change
V11	Minding safety rules when dealing with unknown people	1.09	1.04	0.04	0.160	No significant change
V12	Awareness of risks of danger or violence when going abroad	1.09	1.06	0.03	0.266	No significant change
–	Awareness of human trafficking	1.00	1.00	0.00	n/a	No change (not testable)

As far as the media corpus and the survey instrument used in this study capture *recruitment-stage* representations, i.e., how an individual who might be a trafficker’s associate is first encountered and initially trusted, most instances analysed here cannot be conclusively assigned to one category. Where the underlying media source explicitly reports a trafficking outcome (e.g., confirmed exploitation, prosecution, survivor testimony), this is noted; where it does not, the trafficker is described as posing a *trafficking risk* in the sense of Article 3’s recruitment-stage indicators, without a claim that trafficking, as opposed to fraud or smuggling, was the confirmed outcome.

### 2.3 The reading intervention design

The procedure included several stages. Firstly, conducive psychological and pedagogical conditions were created: the survey was incorporated into online class activities in Critical and Analytical Reading and Stylistics, and the questionnaire was anonymous. Secondly, Part 1 and Part 2 of the survey were conducted sequentially. Thirdly, a simple statistical analysis of the preliminary results in percentages was done to observe the tendencies. After that, the survey data were processed with SPSS 26 for Windows software: the Paired Samples *t-test* was used to measure the pre-reading and post-reading results as one pool of responses.

The sample included 69 B.A. graduates from Borys Grinchenko Kyiv Metropolitan University, who were 91.7% female and 7% male respondents, aged 20–23. They had been studying English for more than 15 years, were reading media regularly (74.5%), predominantly about culture (38.9%), politics (27%), and society (16.7%), and were living in the state of citizenship (95.8%). The study employed a single-group pre−/post-reading repeated-measures design. All 69 participants completed the pre-reading measures and were exposed to the same set of written media materials. They subsequently completed the corresponding post-reading measures. No untreated or alternative-intervention control group was included. The design therefore permits analysis of within-participant change between measurement points but does not provide a counterfactual against which that change can be compared specifically to the reading intervention. At the same time, the questionnaire items contained certain focal points corresponding to the identified variables used to test whether the preexisting vulnerabilities of students remained the same or changed after the respondents read the texts, and to what extent. The questionnaire was designed to answer whether the vulnerabilities could be moderated due to reading activities for social purposes during the educational process in the humanities. The wartime factor was not particularly accentuated in the questionnaire; however, this was an implied condition as students had been under the pressure of the Russia-Ukraine war for more than a year and a half as of the date of surveying.

During the pre-reading survey (Part 1) of the survey, the students answered general and specific questions. The general items included statements about their willingness to move abroad for education and employment opportunities, to seek temporary protection as a war refugee, or migrate for permanent residence, as well as their belief in a successful future career in Ukraine, being in demand in Ukraine, their patriotic feelings, and overall outlook on the future in Ukraine. Media materials used in the reading stage. The specific items included the types of potential traffickers and the level of trust in them when crossing the national border. In this stage, the nominal scale variable (V1) was used to identify which types of potential students had preexisting vulnerabilities to:

- V1: trusting.
  - V1.1. a volunteer at the border crossing.
  - V1.2. a representative of an employment/medical/migration/marriage/travel/education agency, etc.
  - V1.3. a prospective employer.
  - V1.4. a fiancé/fiancée.
  - V1.5. a relative or friend.
  - V1.6. a loving partner.
  - V1.7. a random person who seems kind and willing to help.
  - V1.8. a young man/woman who is willing to help.
  - V1.9. a middle-aged woman who is offering help.
  - V1.10. a middle-aged man who is offering help.

Based on the most frequent responses to the questionnaire used in Part 1, 9 pairs of variables were identified to correspond to the pre-and-post-reading reactions according to the ordinal scale, such as.

- V2: trusting in a volunteer at the border crossing,
- V3: trusting in a representative of an agency (employment, medical, migration, marriage, travel, education agency, etc.),
- V4: trusting in a prospective employer,
- V5: trusting in a relative or friend,
- V6: trusting in a loving partner,
- V7: trusting in a random person who seems kind and willing to help,
- V8: trusting in a young man or woman who is willing to help,
- V9: trusting in a middle-aged woman who is offering help,
- V10: trusting in a middle-aged man who is offering help, and 2 nominal scale variables, such as.
- V11: safety rules dealing with unknown people,
- V12: risks of exposure to danger or violence when going abroad,

Unlike V2–V6, which were paired with fragments constructed around the corresponding role-based type of trafficker, V7–V10 were deliberately not matched to a dedicated fragment. Whereas V2–V6 test whether trust in a specific role (volunteer, agency representative, employer, relative/friend, loving partner) changes after reading a fragment centred on that role, V7–V10 test whether this effect extends beyond the specific roles presented in the reading materials to more general trust judgements based on an individual’s appearance or demographic characteristics such as “a kind stranger, a young attractive person, a middle-aged woman, a middle-aged man”. This design therefore distinguishes between role-specific learning and a broader change in protective judgement that generalises beyond the examples explicitly presented.

The variables V2–V10 were measured on a 4-point ordinal scale with verbal labels (“absolutely not,” “rather not,” “rather yes,” “yes,” coded 1–4), without a neutral midpoint, which was a deliberate design choice to reduce central-tendency bias and to require a directional response on each administration of the item. V1 used a nominal (single-select, multiple-choice) scale across ten types of individuals associated with traffickers, as described above, and V11–V12, along with the two supplementary post-fragment items (“Have you ever thought it is possible?” and “Are you emotionally affected by the story you have read?”), used a binary yes/no nominal scale.

V2–V10 were designed and analysed as nine conceptually distinct single-item measures of trust toward nine different types of individuals who are potentially associated with traffickers, each tested as a paired pre−/post-reading item and independently via paired-samples *T*-tests. These measures were not combined into a single composite score because a respondent may reasonably trust an agency representative while distrusting a stranger. The paired samples design specifically tests whether trust in these types of individuals differs in response to the reading intervention. All nine items used an identical, fixed 4-point verbal format across all variables, which supports comparability of the pre- and post- shift across items. Test–retest reliability was not separately assessed, since the pre−/post-reading design deliberately measures change, which is acknowledged as a limitation in the Discussion.

To establish the level of responses, 8 media text fragments (A–H) were selected, each highlighting the situational characteristics of traffickers, with approximately 1–2 min of reading time^9^. Informative and narrative media texts were used to illustrate the diversity of traffickers’ roles across the identified variables. The requirements for the texts were that they must:

1. be short for reading (from 1 to 3 min each),
2. be representative (for instance, from an NGO’s website) (which is secured by the results of the corpus analysis, meaning that they reflect the conceptual portrait of the trafficker but not a random or occasional description),
3. depict a variety of traffickers (meaning that a range of traffickers must be represented), and.
4. be relevant to the target audience according to the pre-reading stage preliminary results (on false employer, friend, lover, volunteer, etc.).

Part 2 used eight short written media fragments (69–74) selected in response to the patterns of trust identified in Part 1 and to represent different recruitment relationships and stages of trafficking-related risk. The materials included: (A) a confirmed forced-labour case involving recruitment through a fraudulent staffing agency and employer; (B) reporting on traffickers approaching refugees around the Ukrainian border, representing volunteer/stranger recruitment risk; (C) an explanatory account of the *loverboy* recruitment mechanism involving emotional manipulation by an intimate partner; (D) first-person survivor testimony concerning trafficking through an intimate relationship; (E) an intentionally ambiguous case involving exploitation by hosts, used to distinguish trafficking risk from fraud and other forms of exploitation; (F) explanatory material on traffickers’ use of trust-building, isolation and coercion; (G) material describing both organised traffickers and opportunistic exploitation involving friends or family members; and (H) material concerning family, friends and intimate partners in child-trafficking recruitment and the spectrum between deliberate exploitation and poorly informed risky decision-making. The complete fragments, source details, and selection rationale are provided in Supplementary Material.

The materials were deliberately heterogeneous. They included confirmed trafficking cases, recruitment-stage risks, typological explanations, and boundary cases rather than presenting every deceptive or unsafe encounter as legally established trafficking. This distinction was intended to expose participants to the broader continuum of recruitment-related risk while maintaining the conceptual distinction between trafficking, trafficking-adjacent exploitation, and general fraud.

Full documentation of the eight text fragments (A–H) used in Part 2 of the survey is provided in Supplementary Material, including, for each fragment: source publication, publication date, direct URL, word count and estimated reading time, the corpus-derived type of trafficker it was selected to represent (e.g., volunteer, agency representative, loverboy), and a brief rationale tying the selection to the frequency and salience of that type of individual in the corpus analysis reported above. This is provided to allow readers to assess whether each fragment functions as an informative or narrative account consistent with the corpus’s conceptual portrait of the trafficker.

All fragments were presented to respondents in English, consistent with the course’s status as an English-as-a-foreign-language Stylistics module. All fragments were used in their original published English, without translation, since the reading intervention was itself an exercise in English-language stylistic analysis; respondents’ English proficiency of more than 15 years of study was reported as a relevant contextual factor. Before the main study, the fragment set was piloted with eight colleagues who are academic staff and researchers not included in the main student sample. They reviewed the eight fragments for comprehension at the target English-proficiency level and for the potential to cause disproportionate emotional distress, given their trafficking-related content. This pilot did not identify comprehension difficulties or concerns warranting exclusion or substantial revision of any fragment, and the fragment set was used in the main study as piloted. The fragments were instead selected using the four criteria above: brevity, representativeness per corpus results, diversity of trafficker type, and relevance to pre-reading results, and were reviewed by the researcher for tone and content before use, in line with the ethics safeguards described in the Ethics statement.

Not all fragments describe confirmed trafficking cases. Two (A, D) show a full trafficking case, meeting all three legal elements (act, means, purpose). Three (B, C, F) explain general recruitment tactics without following one case to its outcome. Three more (E, G, H) were chosen specifically because they sit on the boundary between trafficking and general fraud or risky-but-non-criminal decisions, rather than treating every deceptive situation as trafficking. This mix was intentional: it avoids teaching students that any deception equals trafficking, in line with the legal distinctions introduced earlier in this paper.

Each fragment was followed by two immediate self-report questions asking whether participants had previously considered the situation possible and whether they were emotionally affected by the story. Across the eight fragments, affirmative responses ranged from 66.7 to 94.5%. These responses provide an indication that participants engaged with the scenarios, but they should not be interpreted as objective evidence that every participant read each text in full or achieved equivalent comprehension. No independent reading-time measure, attention check or comprehension test was included in the original design.

The collected responses were processed with a computer program for statistical analysis (SPSS 26 Windows software) (van Peer et al. 2012: 148), which accurately handles the survey data depending on the research purposes. It provides both descriptive and inferential statistics. A researcher needs to enter data manually and designate independent and dependent variables, analyse the descriptive data of respondents (e.g., age, gender, origin, etc.), explore whether the distribution of responses is normal, and then choose the test for the analysis. Within this research, a parametric test “The Paired Samples *T* Test” (van Peer et al. 2012: 235) was used for *dependent, related, repeated*, and *paired samples* in a *within-subjects* design for testing the pre-reading and post-reading responses to the same questions (van Peer et al. 2012: 231).

## 3 Results

This section outlines the results of each stage of the study. It highlights the findings of the corpus analysis and observations of the media content, the conceptual portrait of the *trafficker* made through frame modelling techniques, as well as the degree of student war-related vulnerability to trusting individuals who may turn out to be traffickers’ associates at border-crossing and their overall migration attitudes established during the pre-reading surveys.

### 3.1 Observations in the media corpus on human trafficking themes

The findings of the corpus analysis indicated the frequent use of lexical units (the number of occurrences is indicated in brackets) used to describe how traffickers gain trust from potential victims in wartime, for example, *Ukraine (335), refugee (225), criminal (127), exploit (104), border (103), vulnerable (96), trafficker (93), war (85), friend (83), vulnerability (78), recruitment (78), pimp (77), love (76), parents (66), trust (65), target (65), agency (56), partner (54), flee (52), a smuggler (45), false (45), cross (40), boyfriend (39), abroad (28), profit (28), intimate (24), migration (23), employer (16), volunteer (15),* and the words with related synonymic meanings. The software’s concordance function allows observation within respective media micro-contexts (see Supplemental Material featuring illustrations extracted from the corpus that outline the most typical characterisation of the trafficker’s roles). These concordances show the media representation of the cases of direct or potential risks of human trafficking in situations of crossing the border of Ukraine under the conditions of increased vulnerability to trusting in alleged traffickers in wartime and general migration flows caused by socioeconomic factors (e.g., the desire for a better life abroad).

### 3.2 Conceptual portrait of trafficker

The conceptual analysis shows the typical representations of *traffickers* as the highest level of generalisation in collective perceptions shaped by the media. The modelling is done through a set of propositional schemas pertinent to five varieties of frames as conceptual structures.

The static (descriptive) characteristics of traffickers are mainly manifested in the propositional schemas of Identification and Comparison Frames. The Identification Frame represents the taxonomy of traffickers through their reference to *individuals* (the *personification* proposition), e.g., *strangers from neighbouring countries*, *members of organised groups* (the *classification* proposition) e.g., *criminals were not slow in exploiting them*, and *seller/middlemen* (the *characterisation* proposition), e.g., *The smugglers who promised them safe passage.* The Comparison Frame represents the schemas of *identity*, *similarity*, and *likeness*. Thus, traffickers are identified as someone who they are, for instance, *smugglers, controllers, recruiters, ex-victims, pimps, corrupted officials*, sometimes *relatives, parents* (e.g., *Case summaries and literature show that parents and siblings may also be directly involved in … trafficking*), Romeo pimps (e.g., *He kept telling me, ‘If you love me, you’ll do this, young, charismatic men who pose as a someone who is interested in dating the victim*), etc.; they are like someone who pretends to be other than traffickers, for example, *a volunteer* (e.g., *The people claiming to be volunteers*)*, a random person who shows a willingness to help refugees, employment/medical/migration/marriage/traveling/education agency* (e.g., *Some traffickers use recruitment agencies as a cover., Labor trafficking within the family unit or an intimate partner relationship can be very difficult to identify*), *loving partner or friend* (e.g., *He kept telling me, ‘If you love me, you’ll do this’*), etc.; while the proposition of similarity is not actuated in this case, which means the absence of figurative language in the depiction of traffickers in the media corpus analysed.

The dynamic characteristics of traffickers are revealed in the propositions of the Action Frame. The *agent-affected* proposition is actuated through representations of actions done by traffickers toward victims, e.g., *[Traffickers] often target [individuals] seeking job opportunities, education, or love, offering false promises to gain their trust*. The *contact* proposition activates the representations of various interactions with potentially vulnerable groups, e.g., *If someone [contacts] you pretending to be a trusted employer interested in hiring new personnel, it may be a scam*. The phrase “it may be a scam” is a lay framing in this case that, depending on subsequent events, may in law amount to either fraud or an act of trafficking under the Palermo Protocol’s means element. The *mode* proposition is actuated through references to individuals or groups who serve as facilitators involved in trafficking or trafficking-adjacent deception, e.g., *The large numbers of unvetted [volunteers] rushing to fill the capacity gap*; using abusive and manipulative practices, e.g., *manipulation of trust, threats, false promises, or a combination of these factors.* The *causation* proposition is actuated through the representation of the vulnerabilities of potential victims and concomitant circumstances such persons may find themselves, e.g., traffickers *prey on the vulnerable and the voiceless*; *The more people who are displaced and the more refugees who migrate across borders, the greater the pool of potential victims of human trafficking*; *For human traffickers, the [war] in Ukraine presents an [opportunity]. The trauma of escaping war, the trajectory of the [conflict in Ukraine], and [uncertainty over their own future] also carry a heavy psychological burden*. As for the *result* proposition, the corpus data refer to profits received by traffickers, e.g., *The case summaries are illustrative of the wide range of [profits] made by traffickers*. The *consequence* proposition is manifested in the language means representing traffickers as being arrested, e.g., *In July 2022, Ukrainian officials [arrested] a Kyiv-based ringleader accused of orchestrating a trafficking ring that sent women recruited on Telegram to work as escorts in Turkey, where they were sexually exploited; Government social workers were present to support the survivors emotionally as the four men who had trafficked them were [arrested]*. Thus, it may be presumed that the concept of TRAFFICKER covers *traffickers, their associates, and others who* act on the victim using threats, violence, false promises of a high-paid job abroad, social media, advertising, acting as wholesalers, pretending to be loving partners, etc. They are shown in the media as those who thrive on the vulnerability of victims and their desperation.

The Romeo pimp/loverboy actor as a type of trafficker identified in the corpus, e.g., “He kept telling me, ‘If you love me, you’ll do this’”, warrants closer treatment than a single frame example, given its prominence in the survey results discussed below. The loverboy method is a documented, staged recruitment process contrary to a single deceptive act: perpetrators who are typically young adult men initiating contact through social media, chat platforms, or in person, deliberately cultivate trust and emotional dependency over an extended period, often through attention, gifts, and declarations of affection, before introducing isolation from family and friends and, subsequently, coercive demands framed as proof of love or as financial necessity within the relationship (31). This staged structure comprising trust-building, emotional dependency, isolation, and coercion explains why the method is comparatively difficult to recognise from the outside and, more importantly, why the person targeted by it is not failing to exercise reasonable caution: the method is specifically designed to be indistinguishable from an ordinary romantic relationship until the coercive stage is already underway. Although the “loverboy” method disproportionately targets young women [(31) reports victims aged 13–25 as the largest affected group], it is documented across genders. Describing it solely as a female vulnerability may overlook male victims and the fact that this form of manipulation is deliberately planned and systematically carried out. Accordingly, this paper treats sustained trust in a romantic partner, as documented in the V6–V10 results, as evidence of the method’s effectiveness as a manipulation tactic; the language used throughout this paper to describe student “vulnerability” refers to exposure to a deliberately engineered risk.

It should be stressed that the Identification and Comparison Frames modelled above operate at the level of *discourse representation*, not legal classification. Not every actor identified through frame analysis meets the threshold of a trafficker under Article 3 of the Palermo Protocol (UN 2000): a volunteer who deceives a displaced person for personal gain without a subsequent exploitative purpose may constitute a case of fraud rather than trafficking, whereas the same deceptive approach followed by coerced labour, sexual exploitation, or forced criminality would satisfy the act-means-purpose structure of the legal definition. The corpus data analysed here document how media discourse constructs the *risk profile* of such actors (volunteer, agency representative, employer, loving partner) without always specifying which cases resolved as trafficking, smuggling, or fraud in the underlying reporting or legal proceedings. However, determining the legal classification of each corpus instance would require case-level judicial evidence beyond the scope of the present study.

### 3.3 Survey results

The preliminary survey held at the pre-reading stage (Part 1) for general feelings about readiness to move abroad and the beliefs in the positive future in the country of citizenship showed no direct vulnerability caused by the economic factor. Students reported their determination to live and work in Ukraine as a country of their citizenship (84.7%), a belief they would be demanded specialists in Ukraine (69.4%) and their knowledge and skills were needed (93%), readiness to contribute to the wellbeing of their country and citizens (79.2%), and an optimistic outlook on their future in Ukraine (61.1.%). At the same time, students were inclined to believe they could earn enough to live comfortably in Ukraine (59.7%), and 91.7% of respondents reported having considered living abroad temporarily. The patriotic feelings of respondents, however, were somewhat balanced against their belief in a better life abroad (52.8%) and having an opportunity to earn more money abroad (90.3%). They showed readiness to use the services of an agency for moving abroad such as a travel agency, migration assistance agency, marriage agency, recruiting agency, etc. (54.2%) having admitted they had thought of emigrating (65.3%) but only 15.3% of respondents planned to do so, while 84.7% denied such plans. Thus, based on the responses to the general items of the questionnaire, it can be inferred that students have a rather strong sense of nationhood and devotion to the country. Nevertheless, by answering the specific items of the questionnaire, students showed their pre-existing trust in alleged traffickers represented by relatives and friends (52.8%), volunteers (6.9.%), employment/medical/migration/marriage/traveling/education agencies (20.8), loving partners (15.3%), and a person who offered to go abroad (45.8%). Thus, the most trusted potential traffickers according to V1 were V1.1. a volunteer at the border crossing, V1.2. a representative of an employment/medical/migration/marriage/travel/education agency, etc., V1.3. a prospective employer, V1.5. a relative or friend, V1.4. a fiancé/fiancée, and V1.6. a loving partner.

In Part 2, students read 8 fragments of media texts selected in response to the pre-existing vulnerabilities revealed in Part 1. The texts highlighted typical situations of human trafficking when victims trusted individuals offering them help before border crossing or after. In this stage, the pairs of responses before and after reading were analysed (V2-V10) according to the ordinal scale (Table 1). As it follows from the table below, the significant statistical result referred to pairs 1–4 and 8, with the following mean values, marked green in Figure 1: [pair 1] before – 2.53, after – 1.98, *p* = 0.000; [pair 2] before – 2.7, after – 2.4, *p* = 0.008; [pair 3] before – 2.56, after – 2.00, *p* = 0.000; [pair 4] before – 3.53 after – 2.84, *p* = 0.000, and [pair 8] before – 1.78 after – 1.55, *p* = 0.04. These values showed significantly reduced levels of trust in volunteers, representatives of agencies (employment, medical, migration, marriage, travel, education agency, etc.), prospective employers, relatives or friends, and middle-aged women willing to help. It should be noted that the survey items (V2–V5) measured a general decline in *trust* in these individuals following exposure to trafficking-themed texts. As the questionnaire did not ask respondents to identify the *type* of harm they anticipated (e.g., exploitation versus financial deception), the reduction of self-reported trust documented here is interpreted as heightened protective caution toward these individuals in the context of moving abroad, rather than as evidence that students specifically came to recognise these individuals as posing a trafficking risk in the legal sense provided above. This distinction is taken up further in the Discussion.

**Figure 1:**
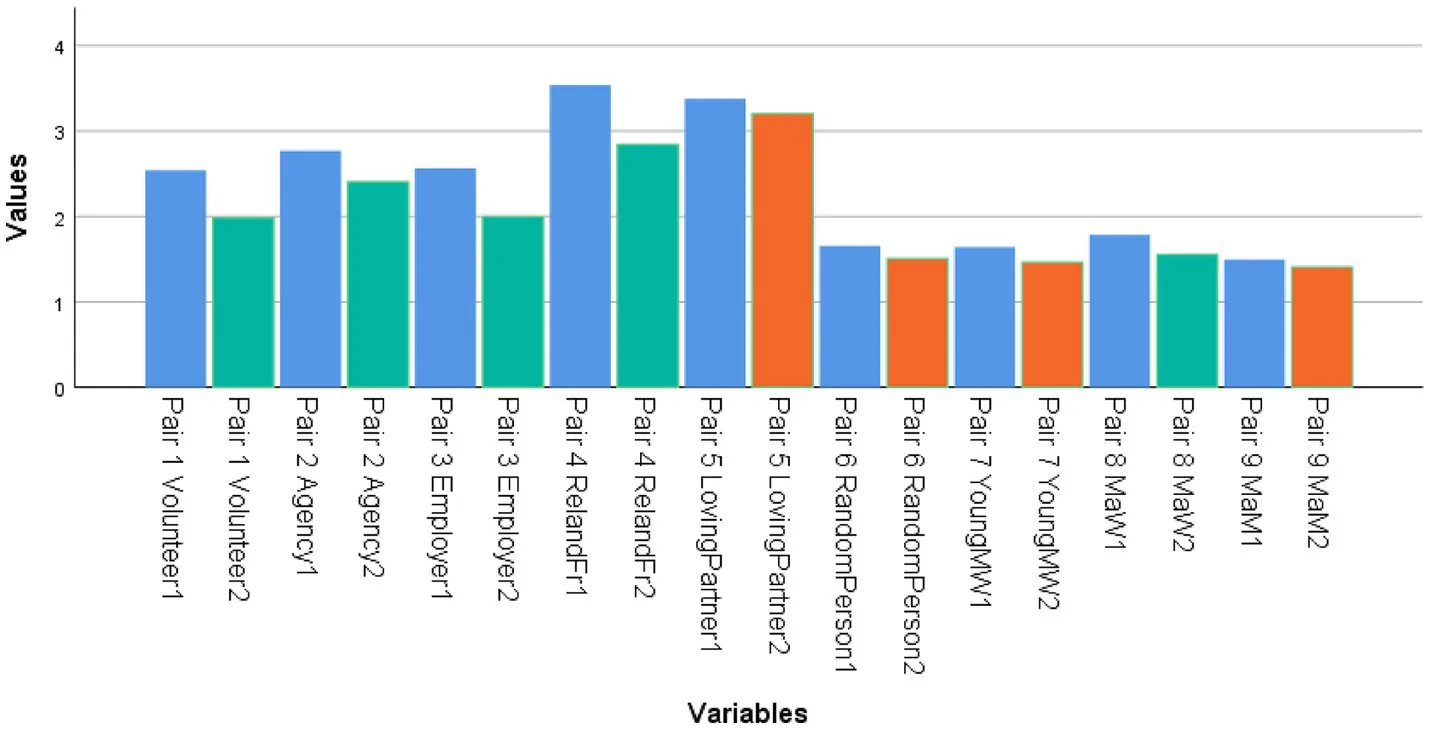
Paired samples *T*-test.

The non-significant statistical results referred to pairs 5–7, and 9, with the following mean values, marked orange in Figure 1: [pair 5] before – 3.37, after – 3.20, *p* = 0.07; [pair 6] before – 1.65 after – 1.50, *p* = 0.12; [pair 7] before – 1.63 after – 1.46, *p* = 0.8; [pair 9] before – 1.49 after – 1.40, *p* = 0.22. These values showed the same level of trust in loving partners or fiancés and random people who seem kind and willing to help, compared with pre-reading presuppositions.

As it follows from the statistical data, no statistically significant pre−/post-reading change was observed for V6–V7 and V9-V10. Within the limits of the present design, these results indicate that participants’ self-reported trust concerning intimate partners and several categories of unfamiliar helpers remained comparatively stable across the two measurement points. They should not be interpreted as evidence that the materials were ineffective; rather, they highlight the need to address the most stable vulnerability to trusting intimate partners in future studies. Two additional variables (pairs 10–11 corresponding to V11–12, respectively) regarding minding safety rules when dealing with unknown people and awareness of the risks of being exposed to danger or violence when going abroad were evaluated on a nominal scale. Mostly, students gave affirmative responses, though the results were non-significant because of the high error probability value (*p*), meaning the absence of a clear tendency across answers. Thus, [pair 10] before – 1.08, after – 1.04, *p* = 0.16; and [pair 11] before – 1.08, after – 1.05, *p* = 0.26.

Compared with the pre-reading data, the following tendency can be seen from the percentage data of V1 in the post-reading stage. The level of respondents’ trust in alleged traffickers represented by relatives and friends increased from 52.8 to 59.8%; volunteers decreased from 6.9 to 2.3%; agencies (employment, medical, migration, marriage, traveling, education, etc.) slightly decreased from 20.8 to 19.5%; and loving partners reduced from 15.3 to 11.5%. Each text fragment suggested for reading was followed by two questions: [*Have you ever thought it is possible?*] and [*Are you emotionally affected by the story you have read?*], the responses to which were [*yes*], ranging from 66.7 to 94.5%. This was indicative of the high involvement of respondents in the situations described in the texts on human trafficking. Despite these high figures, trust in loving partners and unknown persons showing a willingness to help remained high, which entails the necessity of targeted interventions regarding these alleged traffickers. Across all variables, the term “alleged traffickers” is used descriptively to refer to the individuals represented in the source media texts as potential recruiters into trafficking situations. The texts selected for Part 2 depicted trafficking outcomes specifically (see Methods), but the pre- and post- measures registered only a general trust judgement.

## 4 Discussion

This study addresses the issue of early prevention of human trafficking at a university level as incorporated into the humanities and related disciplines. It reveals the potential of using close reading activities to explore the preexisting vulnerabilities to trusting various potential traffickers when moving abroad. The research complements existing studies on violence; for instance, the work by S. Shurma deals with identifying alleged perpetrators (32), as well as the explorations of how conflict situations have been exploited to commit various crimes, including human trafficking, and the impact of warfare on the displaced populations and their growing vulnerability to trafficking (33).

This awareness initiative is aligned with an interdisciplinary paradigm alongside recent works such as public health (34–36), social sciences (37, 38), and raising awareness in medical education (39). It aspires to broaden the social portrait of potential victims by highlighting the vulnerability of the youth currently experiencing war in Ukraine. Similar directions have been recently considered in the works by Al-Tammemi et al. (37), highlighting the sociodemographic profile of victims, and Villacampa (40), dealing with the economic factors aggravating the vulnerability of victims such as “to find a better life”, “distress and desperation”, and “the dream of coming to Europe”, etc.

Another aspect is spreading awareness of various forms of exploitation as primary prevention interventions. In the work by Savoia et al. (41), primary prevention refers mainly to educational efforts directed to raise awareness about the risk and protective factors associated with human trafficking, while secondary prevention is regarded as taking educational action to identify victims (41). As an early prevention initiative, this research relies on the synthesis of Pedagogical Stylistics (28, 42–45) and social studies that justify the approach of teaching stylistics for social purposes. The awareness of students about the most typical alleged traffickers can foster more mindful decision-making for temporary or permanent living abroad due to the war against Ukraine. This educational primary prevention effort can be achieved through informative and narrative media texts to stimulate empathy and tolerance and reduce the stigmatisation of vulnerable social groups. Through the representations of traffickers as characters in a media narrative, the audience can develop an understanding of typical traffickers, thus avoiding erroneous decisions. The socio-oriented teaching of humanities extends the horizons of literary education in stylistics supported by empirical studies (27, 46, 47) and a corpus-based approach to reader response (48). The targeted “attention-directed” [(27): 169] exposure to texts on socially relevant topics also raises media-linguistic awareness through critical reading and helps moderate social behaviours (65).

At the same time, the distinction the mentioned studies draw between primary prevention (awareness) and secondary prevention (identification of victims) maps onto the distinction between the prevention and protection pillars of the 3P/4P framework. A rights-based extension of the curriculum would therefore pair the reading-based awareness activities piloted here with concrete referral information, for instance, incorporating the contact details and referral pathway of Ukraine’s National Referral Mechanism or the relevant NGO/hotline structure into the course materials themselves, following the model of National Referral Mechanisms developed by the OSCE Office for Democratic Institutions and Human Rights, which coordinate state and civil-society actors to identify, protect, and refer trafficking victims (49). Embedding a referral pathway alongside the literary and linguistic material would shift the intervention from awareness alone beyond pedagogical settings toward a safe identification and referral function.

The pedagogical intervention piloted in this study is done within a single pillar of a much wider international framework for combating trafficking, conventionally summarised as the “3P” (prevention, protection, prosecution) or, since 2009, “4P” paradigm, which adds partnership as a fourth, cross-cutting pillar (50). Reading-based awareness activities of the kind developed here are pertinent to primary prevention; they neither can substitute for nor should be understood as reducing the urgency of the state’s protection obligations (identifying and supporting victims), prosecution obligations (investigating and punishing traffickers), or the partnership structures that connect educational institutions to the referral systems responsible for the other three pillars. This distinction is important because a human rights-based approach to trafficking requires more than awareness: the OHCHR Recommended Principles specify that anti-trafficking measures must be non-discriminatory in their effect on the rights of trafficked persons and other groups, including migrants, internally displaced persons, and asylum seekers [(18), Principle 3], and that trafficked persons must have access to effective and appropriate remedies [(18), Principle 17]. For the population addressed in this study, who are university students displaced or made vulnerable by the Russia-Ukraine war, these principles are relevant as many respondents fall within categories (IDPs, refugees, temporary protection seekers) whose protection needs already engage separate international frameworks. Any prevention curriculum operating in this context should be explicit that it is a complement to, not a substitute for, formal protection and referral systems.

It should be stated that the vulnerability patterns discussed throughout this paper, such as students’ trust in volunteers, agencies, employers, relatives, or romantic partners, are described here as targets for educational intervention and do not imply characteristics attributable to the students themselves. The outcomes concern self-reported perceptions rather than observed behaviour. Reduced willingness to trust a hypothetical volunteer, agency representative, employer or other actor does not demonstrate that a participant would identify or avoid an actual trafficking attempt. Nor does a decrease in general trust necessarily indicate accurate recognition of trafficking in the legal sense, since some of the scenarios intentionally occupied the boundary between trafficking risk, exploitation and fraud. The results therefore concern one limited component of trafficking-related vulnerability, which is protective caution in self-reported trust judgements. Under a due diligence standard, the responsibility to prevent trafficking rests primarily with the state and with the institutions and individuals who exploit positions of trust. The pedagogical aim of this research is to expand students’ interpretive resources for recognising risk, in addition to the structural and legal protections that remain the state’s obligation to provide.

It is important to note that the Paired Samples *T*-test findings reported above (V2–V5) document a statistically significant pre−/post-reading change in self-reported trust toward specific types of individuals who may be involved in human trafficking, measured immediately after a single reading intervention. This is a measured change in attitude and stated belief; however, it is not a measure of behaviour, of retention over time, or of trafficking incidence, and the study makes no claim of the latter kind. The “primary prevention” intervention should therefore be understood in the same sense used by Savoia et al. (41), i.e., as educational efforts that raise awareness of risk and protective factors, though no claim is made that the intervention reduces the occurrence of trafficking, which this design cannot demonstrate and does not attempt to.

A question may arise as to why self-reported trust should be treated as a meaningful indicator of trafficking vulnerability instead of an unrelated attitudinal measure. Trust plays a specific role in the documented mechanics of trafficking recruitment. Empirical accounts of recruitment consistently describe trust-building as a deliberate, time-extended tactic that precedes exploitation, rather than an incidental feature of it: traffickers are documented as spending weeks or months cultivating a relationship of trust with a prospective victim before any coercive or exploitative move is made (51). Reviews of vulnerability measurement in the trafficking literature similarly treat interpersonal trust and relational vulnerability as recurring, if imperfectly operationalised, components of composite vulnerability indicators, alongside socioeconomic and demographic factors (52). On this basis, a measurable shift in how readily a respondent extends trust to the types of individuals most frequently implicated in recruitment narratives (volunteers, agencies, employers, romantic partners) can be interpreted as an indicator of one recognised component of trafficking vulnerability, which is susceptibility to trust-based recruitment, and does not imply overall trafficking risk or trafficking prevalence.

This distinction explains how the V6–V10 null results should be interpreted. The absence of significant change in trust toward loving partners and “kind strangers” is not evidence that these relationships carry lower trafficking risk. The recruitment literature suggests the opposite: romantic-partner or so-called “loverboy” recruitment is a well-documented high-risk pathway (51). These results show that a single short reading intervention does not shift an attitude that may be more deeply entrenched or more emotionally resistant to revision than trust in institutional individuals, which opens a perspective for further studies with more targeted intervention design. This resistance is consistent with the staged, trust-dependent mechanics of the “loverboy” method described above (see Conceptual portrait of trafficker): because the method is designed to sustain emotional trust even as coercive control develops, a single reading intervention addressing recruitment tactics may be less effective at shifting trust in an active or hypothetical romantic relationship than at shifting trust in more transactional individuals such as agencies or employers. This suggests that future interventions targeting the “loverboy” pattern specifically may require narrative or scenario-based materials that model the staged progression of the method instead of general trafficking-awareness content.

## 5 Limitations

The limitation concerns the nature of the indicators available in a media-text corpus, as distinct from the indicators used operationally to identify trafficking on the ground. Field-oriented frameworks such as the ILO’s Operational Indicators of Trafficking in Human Beings (30) identify indicators across six stages of the trafficking process: deceptive recruitment, coercive recruitment, recruitment by abuse of vulnerability, exploitation, coercion at destination, and abuse of vulnerability at destination, many of which (e.g., confiscation of identity documents, restricted movement, withheld wages, physical or psychological control at the destination) are only observable after a person has already entered an exploitative situation, typically through direct, proximate contact rather than through third-person narrative (53). Public-facing “red flag” lists [e.g., (53)] similarly distinguish *recruitment-stage* warning signs such as implausible job offers, unsigned or foreign-language contracts, upfront fees, and asymmetric romantic relationships from *destination-stage* indicators of actual exploitation, which require sustained observation of a person’s living or working conditions.

The media corpus analysed in this study is limited to the first category. Journalistic and NGO texts, almost by genre convention, foreground the recruitment-stage narrative, i.e., how trust was gained and by whom, because this is what is reportable, quotable, and comprehensible to a general media audience, while destination-stage exploitation is typically reported only in retrospective summaries. This means the conceptual portrait of “the trafficker” developed in this paper and the media fragments used in the survey necessarily emphasise recruitment-stage red flags that might point to elevated risk rather than confirmed trafficking. The same recruitment-stage cues such as an unusually generous offer, an unfamiliar agency, or an accelerated romantic relationship can equally precede a fraud that never escalates to exploitation, a smuggling arrangement that concludes without incident, or a legitimate opportunity. Consequently, the pedagogical intervention piloted here should be understood as training students to recognise recruitment-stage caution cues associated with elevated risk of trafficking, smuggling, and fraud alike and not as training them to diagnose trafficking as opposed to other outcomes, which would require ground-level indicators such as control of documents, movement, and wages; signs of coercion, which are not represented in recruitment-stage media narratives.

A broader limitation concerns causal inference. The study was done within the reader-response linguistic pedagogical paradigm. The findings should be interpreted within the constraints of a single-group pre−/post-reading design. All participants received the same materials, and no control or comparison group was included; therefore, the study does not provide a counterfactual showing how participants’ responses might have changed in the absence of the reading activity. The paired-samples analysis establishes whether responses differed between the two measurement points within this sample, showing students’ readiness to mind safety rules when dealing with unknown people and the risks of being exposed to danger when abroad. However, it does not show whether the change persisted or translated into protective behaviour, as the statistically significant reductions observed for V2–V5 are interpreted as short-term changes in self-reported trust associated with the pre−/post-reading sequence and not as causal evidence of intervention effectiveness. Future research will include a control or delayed-intervention group, a follow-up period, and at least one behavioural or intention-based measure, for instance, verification intent before accepting an offer of help, willingness to consult a hotline or referral service, or subsequent actions that would connect the attitudinal shift documented here to an actual protective behaviour. The present study should therefore be viewed as a pilot demonstrating feasibility and short-term change, rather than as a validated prevention intervention.

A second limitation concerns intervention fidelity. Although immediate self-report questions and affirmative responses followed each media fragment indicated substantial reported engagement, the study did not independently verify that every participant read every fragment in full or understood it to the same degree. In this regard, the study relies on the fact that English humanities students have been in linguistic environments throughout their studies. Evidence indicates that individuals processing a narrative in their second language feel just as transported into the story as those processing the same narrative in their native language (54). Therefore, no attention checks or comprehension tests were meant to be done to determine how variation in exposure or comprehension contributed to individual response changes.

A related limitation concerns the composition of the pilot group for the fragment set: the eight colleagues who reviewed the fragments for comprehension and emotional impact before the main study were academic staff and researchers, not members of the target student population, and not individuals directly affected by wartime displacement in the way much of the main sample was. While this pilot provided a useful check on readability and tone, it cannot fully substitute for piloting with peers of the actual target population, whose reception of trafficking-related content, particularly given the sample’s own proximity to displacement, may differ from that of the pilot group. Future iterations of this research would benefit from piloting with a small, appropriately supported group drawn from the target student population itself.

Finally, the generalisability of these findings is bounded by the sample’s specific composition. All respondents were from a single institution (Borys Grinchenko Kyiv Metropolitan University), limiting confidence that the results extend to students at other Ukrainian universities with different regional, socioeconomic, or demographic profiles. The sample was also overwhelmingly female (91.7%), which reflects the gendered composition of the university’s B.A. cohort in this programme rather than a deliberate sampling choice, but means the findings speak more directly to female students’ trust patterns than to those of male students, who are independently subject to different mobility constraints under wartime martial law (see the Introduction). Also, the study was conducted at a specific moment in an ongoing war, among students who had lived under wartime conditions for more than 18 months; both the direction and magnitude of the reported effects may not generalise to peacetime contexts, to earlier or later phases of the war, or to displaced populations outside formal higher education. Considering that only 7% of the sample was male, this study cannot examine sex-based differences in trafficking-related trust vulnerability. Although the Introduction notes that women are disproportionately represented among displaced populations and among those targeted by traffickers (1), the sample is too imbalanced to test or extend this observation. Addressing sex differences would require a deliberately balanced, multi-institutional sample and is therefore identified as a direction for future research.

## 6 Conclusion

The results of the study shed light on the students’ perceptions of potentially dangerous individuals who might act as trafficking facilitators or as perpetrators of trafficking-adjacent fraud and their presuppositions of trusting in them while moving abroad in wartime. The conducted corpus and frame analyses helped reveal the recurrent types of traffickers in media and identify the types of potentially dangerous individuals described in media, which, in turn, helped select tailored media fragments for the reading intervention.

The alternative hypothesis was partially proved regarding V2-V5: trust in volunteers at the border crossing, representatives of agencies (employment, medical, migration, marriage, travel, education agency, etc.), prospective employers, and relatives or friends. After students read the fragments of texts suggested in Part 2 of the survey, they changed their minds, showing significantly lower levels of trust in the stated types of alleged traffickers. The null hypothesis also proved partially true regarding V6-V10: trust in loving partners or fiancés, and random people who seem kind and willing to help, including young or middle-aged men or women willing to help. The perceptions of alleged traffickers did not change after the students read the fragments of texts used in the reading intervention. These lasting and stable vulnerabilities uncover the necessity to explore the means of targeted impact on students’ perceptions of the image of a “loverboy” in media and survival narratives and elaborate on the profile of this type of trafficker corresponding to the variable – trusting in loving partners.

Future studies should strengthen intervention fidelity by incorporating objective attention and comprehension checks. They could also compare written media narratives with multimodal prevention materials, including short survivor-centred videos, simulated fraudulent job advertisements, social-media recruitment messages and interactive red-flag identification tasks. Such designs would make it possible to test whether they can accurately identify specific recruitment tactics and distinguish trafficking indicators from general fraud or unsafe migration situations.

Another prospective study refers to the linguistic markers of dealing with alleged traffickers in social media communications, which will reveal the typical words and phrases used by human trafficking manipulators in correlation with the stable vulnerability of trusting loving partners and random people willing to help, considering the existence or absence of the wartime factor. A further priority for subsequent research is a deliberately sex-balanced or multi-institutional cohort, designed specifically to test whether the vulnerability patterns identified here and their responsiveness to the reading intervention differ meaningfully between male and female students, which the present sample’s composition does not allow us to assess. These findings are offered as a contribution to the prevention pillar of anti-trafficking policy. They should not be read as suggesting that individual vigilance can substitute for the protection, prosecution, and referral obligations that remain the state’s responsibility under international human rights law.

## Data Availability

The original contributions presented in the study are included in the article/Supplementary material, further inquiries can be directed to the corresponding author.

## Footnotes

1 Regarding migration, sociological studies show that Ukrainian citizens have always striven for a free movement of persons (8, 55–57) and shared EU values and practices reliable information could create or intensify opportunities for trafficking and exploitation (63, 66, 68).

2 Some of the media-reported cases can be referred to the publications by Hill (58), Hughes (59), White (60), Kelly et al. (61), Piña (62), and Van (75).

3 This paper’s use of “good Samaritan” throughout refers specifically to this deceptive performance and should not be read as casting suspicion on the large majority of genuine volunteers and hosts who provided legitimate assistance to displaced people during the war; the distinction between the two is precisely what the frame-analytic model developed below (see Conceptual portrait of trafficker) is intended to help readers draw.

4 The study is carried out in teaching the Stylistics of the English Language as a foreign language as part of the philological specialties at Borys Grinchenko Kyiv Metropolitan University. The research is a part of the initiative *Changing Young Minds: Student Awareness of Human Trafficking under War Conditions* implemented with the support of the French Research Center in Humanities and Social Sciences (CEFRES) in Prague, the Czech Republic, in the framework of the non-residential fellowship program “CEFRES Actions for Ukraine” CEFRES, UAR 3138 CNRS–MEAE, and covers a series of interdisciplinary studies responding to increased human trafficking risks in an educational setting. The objective of the project is to collect empirical evidence on how human trafficking is perceived by young people studying at a Ukrainian university and establish the cognitive and sociolinguistic prerequisites for creating impactful anti-trafficking educational products.

5 [ske.li/trusted_traffickers].

6 The annotation strategy covered the representations of the trafficker’s role which, apart from the direct nominations (trafficker) may also include the words parents, boyfriend, loving partner, employer, etc., which in other contexts may have meanings other than that of “trafficker.” Therefore, when analysing the language corpus, only those contexts actuating the semantic roles of “traffickers” were regarded in this research.

7 The notion of a *proposition* means the most basic unit of meaning in a representation (*University of Alberta Dictionary of Cognitive Science: Proposition*, n.d.).

8 The terms *qualitative (what kind of?), quantitative (how much/many?), locative (where?), temporative (when?)*, and *mode of existence (how?)*, etc. are given in the wording of Pr. S. Zhabotynska’s terminology (29).

9 Approx. 11 min 07 s of total reading time as estimated on https://niram.org/read/
