# Supplementary material for: Trusted traffickers and prosocial humanities: exploring Ukrainian student vulnerabilities to wartime cross-border risks
Source: Front Public Health. 2026 Oct 30;14:1818656. doi: 10.3389/fpubh.2026.1818656 (PMC13760692; doi:10.3389/fpubh.2026.1818656)
Supplement: Supplementary file 1 [file Data_Sheet_1.pdf]

frontiers

Supplementary Material

1 Supplementary Data

1.1. Language data

The findings of the corpus analysis (Paliichuk, E. (2023). *ske.li/trusted_traffikers* [Dataset]. Available at: https://ske.li/trusted_traffikers available as of 2023) indicated the frequent use of lexical units (the number of occurrences is indicated in brackets) used to describe how traffickers gain trust from potential victims in wartime, for example, *Ukraine (335), refugee (225), criminal (127), exploit (104), border (103), vulnerable (96), trafficker (93), war (85), friend (83), vulnerability (78), recruitment (78), pimp (77), love (76), parents (66), trust (65), target (65), agency (56), partner (54), flee (52), a smuggler (45), false (45), cross (40), boyfriend (39), abroad (28), profit (28), intimate (24), migration (23), employer (16), volunteer (15),* and the words with related synonymic meanings. The software's concordance function allows an observation within respective media micro-contexts. Some illustrations extracted from the corpus outline the most typical characterisation of the trafficker's roles. The keywords are taken into square brackets.

2. *For instance, a [trafficker] may lure a victim with promises of a loving relationship and gifts while working to isolate and manipulate the victim.*
3. *In this scenario, once the [trafficker] manages to gain the [trust] of the victim and isolate them, the [trafficker's] 'loving' attitude switches to reveal their real personality and motives.*
4. *The [trafficker] now may fully engage in abusive tactics – physical, psychological, emotional, or mental – to exert his or her control over the victim (yes, a trafficker can be a "her") to whatever ends the [trafficker] desires.*
5. *In some cases, a [trafficker] may manipulate or construct a situation or circumstance to create an artificial debt for the victim to work off.*
6. *Once united with the [trafficker], communication will be restricted, and threats or punishments will be put into place to further entrap and isolate the victim.*
7. *[Pimps] posed as someone else, gained the [trust] of their victim, made promises of false opportunities, and relocated their victims away from their homes to be forced to perform repeated commercial sex acts for the profit of the [trafficker].*
8. *Women and girls, often poverty-stricken or desperate for work, may come across job offerings online, where a [trafficker] will typically promise an often "unbelievably" good job, only to ensnare the victim into sex slavery once relocated.*
9. *Ensnaring victims typically involves manipulation of [trust], threats, false promises, or a combination of these factors.*
10. *This results in an enormous power imbalance, a carefully crafted false sense of [trust] to lure in victims, and a built-in escape for the [trafficker] in case suspicions arise (as only they control what is known about their true identity and whereabouts).*
11. *Since [trust] is a vital piece of this particularly manipulative process, the [trafficker] tends to place emphasis on wanting to help the victim get out of difficult circumstances or offering a*

new opportunity for work or fame, but with the motivation of helping the victim as someone they care about.

12. *Of course, this [trust] relationship is [exploited] only as a means of obtaining a new sex worker to profit from.*
13. *In both cases, the theme of [exploiting] the [trust] of a vulnerable girl can be found.*
14. *"I think the mindset is what eventually starts out as [trust] and hope becomes fear and . . . sometimes just what we call a flat effect where there's no feeling anymore," said Loyie.*
15. *Then, while having to [trust] strangers from neighboring countries, these individuals, especially unaccompanied minors and women, are at risk for deceptive offers from [traffickers]."*
16. *It could be someone they are "dating" when in reality that individual was building [trust] and grooming them to be trafficked for sex.*
17. *In some cases, the [traffickers] are their own [family members].*
18. *I [trusted] my [boyfriend] and I went to live with him, but he lied to me.*
19. *By masking their true intentions and presenting themselves as [trust]worthy individuals, [traffickers] exploit the victims' [trust] and lead them into exploitation.*
20. *[Traffickers] often [target] individuals seeking job opportunities, education, or love, offering false promises to gain their [trust.]*
21. *If someone contacts you pretending to be a [trusted] [employer] interested in hiring new personnel, it may be a scam.*
22. *This creates a situation where the worker becomes dependent on the [employer] for food and shelter," the ILO explains.*
23. *A would-be [employer] collects fees from a potential worker for the "opportunity" to work in a particular job.*
24. *Domestic workers may or may not live in their [employer]'s homes.*
25. *Some victims are trafficked by a [parent], [boyfriend /girlfriend], or [people they consider friends].*
26. *Her [boyfriend], a man she loved and [trust]ed, sold her to another man who took her to Italy.*
27. *I want my story to educate you on the [boyfriend] tactic.*
28. *In another case, a young woman was trafficked by her [boyfriend] who was in the military.*
29. *Inside secret army of [volunteers] smuggling Ukrainian families over … border.*
30. *In March 2022, EUROPOL issued an early warning notification calling for heightened attention to be paid to [border crossing] points, reception areas, and transit and destination hubs in the EU.*
31. *The large numbers of unvetted [volunteers] rushing to fill the capacity gap were a particular concern: … 'while the generosity and solidarity towards Ukrainian refugees have been inspiring, states must prevent predatory individuals and [criminal] networks from [exploiting] the situation'.*
32. *This assessment corresponded with reported incidents in the early days of the war of refugees being [targeted] by individuals offering food, accommodation, or transport who would then attempt to [exploit] the [refugees].*
33. *The people claiming to be [volunteers] announced that [refugees] who don't have a hard copy of their passports should make themselves known to be "sorted" before reaching the stamp point and they were then placed into a separate line, the witness said.*

34. *Case summaries and literature show that [parents and siblings] may also be directly involved in … trafficking.*
35. *Have a controlling [parent], [guardian], [romantic partner], or ["sponsor"] who will not allow them to meet or speak with anyone alone or who monitors their movements, spending, or communications.*
36. *Educators may also observe potential indicators of human trafficking in the [parents], [family members], and [caregivers] of their students.*
37. *Often, they fall into the category of ["Romeo pimps"] – young, charismatic men who pose as a someone who is interested in dating the victim.*
38. *Is in the commercial sex industry and has a controller [boyfriend, pimp, manager, or "daddy"] or mentions having … an overly controlling or abusive [boyfriend]*
39. *The letter calls on the countries of asylum to train frontline groups working with [refugees] on the signs of potential trafficking; to implement systems that allow safe and legal routes for [refugees]...*
40. *Attempting to reach safety, many [refugees] resort to the services of [smugglers].*
41. *A British news correspondent warns of the vulnerable state of [Ukrainian] [refugees], describing a scenario in which a mother and son were robbed of all their valuables and documents by a driver who offered them assistance in Romania, a country that has received more than 80,000 refugees so far, has confirmed the prevalence of human trafficking of [Ukrainian] [refugees].*
42. *Where a [refugee] crisis prevails, there is a strong likelihood that so too does human trafficking.*
43. *Labor trafficking within the family unit or an [intimate partner] relationship can be very difficult to identify …*
44. *The [smugglers] who promised them safe passage turn around and demand more payment.*
45. *Many interviewees said [smugglers] were cheaper and quicker than regular [migration], the report found.*
46. *Some [smugglers] also took advantage of the provisions of temporary protection to help smuggle third-country nationals into Europe in 2022. … This in turn provided an opportunity for [smugglers] to bring new clients into Ukraine, as they could then merge these clients into refugee flows into Europe.*
47. *One member of staff at the IJM, who is working close to the [Ukraine]-Romania border in shelters housing [refugees], told The Independent that people [smugglers] were known to be [targeting] people, including women and children, once they have [crossed the border].*
48. *For those who have stayed, however, the war brought opportunities – and [criminals] were not slow in exploiting them.*
49. *For human [traffickers], the [war] in [Ukraine] presents an opportunity.*
50. *The trauma of escaping [war], the trajectory of the conflict in [Ukraine], and uncertainty over their own future also carry a heavy psychological burden.*
51. *The more people who are displaced and the more [refugees] who [migrate] across [borders], the greater the pool of potential victims of human trafficking.*
52. *Some [traffickers] use [recruitment agencies] as a cover...*
53. *He kept telling me, 'If you [love] me, you'll do this.*
54. *[Traffickers] often [target] individuals seeking job opportunities, education, or [love], offering false promises to gain their [trust].*

55. *For those willing to sacrifice the basic human rights and dignities of others to turn a [profit], the sex trafficking industry is tempting indeed.*
56. *Nor does it tackle the interests–and [profits] –that drive trafficking.*
57. *Finally, [traffickers] often [prey on extremely vulnerable individuals] such as children or adolescents ensnaring them with promises of love and material resources.*
58. *[Traffickers] are experts at [exploiting] victim [vulnerabilities] and finding ways to make victims dependent in some way.*
59. *[Traffickers] identify and exploit these [vulnerabilities], trapping victims in situations of [exploitation] from which escape seems impossible.*

The illustrated concordances show the media representation of the cases of direct or potential risks of human trafficking in the situations of crossing the border of Ukraine under the conditions of increased vulnerability to trusting in alleged traffickers in wartime and general migration flows caused by socioeconomic factors (e.g., the desire for a better life abroad).

1.2. Selected media used in the experiment

Fragment A. Figure-type: agency representative/employer (V3/V4). Risk category: trafficking risk, confirmed outcome. Source: Williams, A., "Human trafficking: an American tradition of silence," *KEYE*, 25 Jul 2023. URL: https://cbsaustin.com/news/armstrong-army-strong/human-trafficking-an-american-tradition-of-silence. This fragment satisfies the full act–means–purpose structure: recruitment via a false employment offer (act), fraud and deception through a fabricated staffing agency (means), and forced labour exploitation as a maid and cleaner (purpose). It reaches a confirmed trafficking outcome, ending with a law enforcement raid, which anchors V3/V4 in an unambiguous case.

Fragment B. Figure-type: border-crossing volunteer/stranger (V2, V7). Risk category: trafficking risk, recruitment-stage and general. Source: Border, A.B.O.T.U., "Ukraine war: human traffickers preying on refugees fleeing conflict," *Sky News*, 19 Mar 2022. URL: https://news.sky.com/story/ukraine-war-human-traffickers-preying-on-refugees-fleeing-conflict-12569986. This fragment depicts recruitment-stage trafficking risk at the border-crossing context directly relevant to this study's population, without following a single case to its outcome. It was selected to instantiate V2 and V7 as elevated-risk recognition material, matching respondents' own displacement experience.

Fragment C. Figure-type: loving partner/loverboy (V6). Risk category: trafficking risk, typological. Source: "How does human trafficking happen?," Hagar New Zealand, 25 Jul 2022. URL: https://hagar.org.nz/human-trafficking-how/. This fragment describes the loverboy method as a general mechanism, covering both the means (emotional manipulation and coercion) and the purpose (sexual exploitation) elements of the legal definition. It was selected to instantiate V6 with mechanism-level content, grounding the loverboy discussion developed later in the paper.

Fragment D. Figure-type: loving partner/fiancé (V6). Risk category: trafficking risk, confirmed outcome. Source: "Human trafficking victim shares story," U.S. ICE, 5 Oct 2021. URL: https://www.ice.gov/features/human-trafficking-victim-shares-story. This fragment satisfies the full act–means–purpose structure through first-person survivor testimony, including the coercive pressure captured in the phrase "if you love me, you'll do this." It serves as the case-level counterpart to Fragment C and is the direct source of the loverboy corpus quote already cited in the Results section.

Fragment E. Figure-type: volunteer/host (V2), with secondary relevance to relative/friend (V5) and loving partner (V6). Risk category: boundary case, trafficking risk versus general fraud risk, left explicitly unresolved. Source: “She left Ukraine and hoped her Canadian hosts would help her start a new life. Instead she faced a new nightmare,” Human Trafficking Search, n.d. URL: https://humantraffickingsearch.org/resource/she-left-ukraine-and-hoped-her-canadian-hosts-would-help-her-start-a-new-life-instead-she-faced-a-new-nightmare/. The source article itself states that exploitation exists “on a spectrum… not all of which crosses the line into criminality,” and the case is not narrated to a confirmed legal outcome. It was selected deliberately as an ambiguous case, useful for illustrating the trafficking-risk/fraud-risk distinction developed in the Methods, rather than reinforcing an assumption that every deceptive approach constitutes trafficking.

Fragment F. Figure-type: general typology, with no single dedicated variable. Risk category: trafficking risk, means element, typological. Source: “The Anatomy Of Human Trafficking,” Malyk, M., EasyLlama Blog, 22 Feb 2023. URL: https://www.easyllama.com/blog/how-to-identify-human-trafficking-perpetrators/. This fragment explains the means element of the legal definition directly, covering trust-building, isolation, and coercion, without reference to a specific case. It was selected as orienting content, establishing the general recruitment mechanism before the role-specific fragments are encountered.

Fragment G. Figure-type: relative/friend (V5), with general typological relevance. Risk category: trafficking risk and general fraud/opportunistic exploitation, spanning a spectrum of intent. Source: “Who Are The Human Trafficking Perpetrators?,” Malyk, M., EasyLlama Blog, 22 Feb 2023. URL: https://www.easyllama.com/blog/how-to-identify-human-trafficking-perpetrators/. This fragment distinguishes organised traffickers from "amateur" facilitators, including friends or family members who exploit an opportunity for money, drugs, or favours without belonging to a criminal network. It was selected because this distinction mirrors the trafficking-risk/fraud-risk boundary discussed in the legal framework subsection, and its ground-level “red flag” indicator list also supports the recruitment-stage/destination-stage discussion in the Limitations.

Fragment H. Figure-type: relative/friend (V5). Risk category: spans trafficking risk through to non-criminal risky decision-making. Source: “Family members are involved in nearly half of child trafficking cases,” Counter-Trafficking Data Collaborative / IOM, n.d. URL: https://www.iom.int/sites/g/files/tmzbdl486/files/our_work/DMM/MAD/Counter-trafficking%20Data%20Brief%20081217.pdf. This fragment explicitly frames family, friend, and intimate-partner involvement in recruitment along “a spectrum of intent,” ranging from cases where exploitation is the deliberate purpose from the outset to cases where families “unwittingly” place children at risk through well-intentioned but poorly informed decisions. It was selected as the fragment that most explicitly operationalises the intent/consent parameter distinguishing trafficking risk from non-criminal, structurally-driven vulnerability, as discussed in the Methods.

2 Supplementary Figures and Tables

3 Supplementary Figures

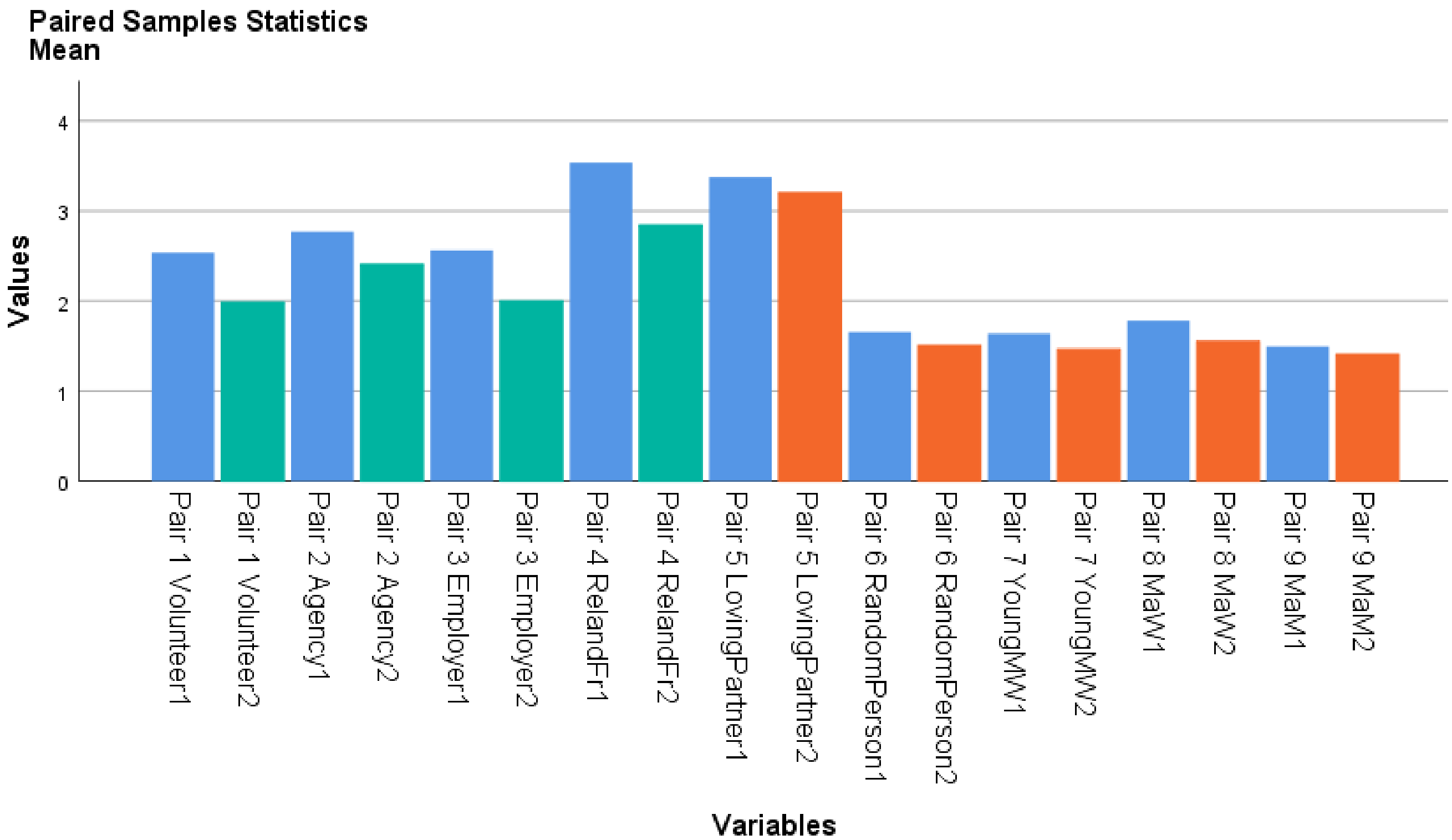

Figure 1. Paired Samples *T*-Test

Table 1. Differentiating parameters across risk categories

**Parameter**	**Trafficking risk**	**Smuggling risk**	**General fraud risk**
Exploitation purpose	Required (defining element)	Absent	Absent
Consent	Legally irrelevant once the means element is present	Freely given	Vitiated re: the deception only
Cross-border element	Not required	Required	Not required
Relationship after initial contact	Ongoing control/coercion	Ends on arrival	Ends once deception is discovered
Non-compliance signal	Victim cannot leave, is not paid, is monitored	Migrant is left to proceed independently	Money/service is not delivered as promised
